# The Role of Apoptosis Pathway in the Cytotoxicity Induced by Fresh and Aged Zinc Oxide Nanoparticles

**DOI:** 10.1186/s11671-021-03587-y

**Published:** 2021-08-09

**Authors:** Juan Wang, Lei Wang, Wenting Zhao, Na Yu, Meiling Cheng, Mingqin Su, Jian Hu, Xiaoyan Wu, Hua Du, Meimei Wang

**Affiliations:** 1 Department of Pathophysiology, School of Basic Medical Science, Anhui Medical University, No. 81, Mei-Shan Road, Hefei, 230032 Anhui People’s Republic of China; 2 MOE Key Laboratory of Population Health Across Life Cycle, No. 81, Mei-Shan Road, Hefei, 230032 Anhui People’s Republic of China; 3 High Magnetic Field Laboratory, Key Laboratory of High Magnetic Field and Ion Beam Physical Biology, Hefei Institutes of Physical Science, Chinese Academy of Sciences, Hefei, Anhui People’s Republic of China; 4 Department of Physiology and Biophysics, Miller School of Medicine, University of Miami, Miami, FL USA

**Keywords:** Aged zinc oxide nanoparticles, Cytotoxicity, Mechanism, Apoptosis, Transcriptomics

## Abstract

Zinc oxide nanoparticles (ZnO NPs) are used in a wide range of applications including industry, commercial products and medicine field. Numerous mechanistic studies for ZnO NPs’ toxicity were performed on pristine (fresh) NPs. However, the cytotoxicity induced by the transformed (aged) ZnO NPs and the underlying mechanisms remain unclear. Here, we observed the physicochemical transformation of ZnO NPs underwent over time, followed by evaluating the cytotoxicity of fresh and aged NPs. We found that fresh ZnO NPs induced higher apoptosis level than their aged counterparts. Accordingly, RNA sequencing data from aged ZnO NP-treated human–hamster hybrid (*A*_L_) cells showed that p53, PI3k–Akt, FoXO, Glutathione, ErbB, HIF-1, Oxytocin and Jak-STAT signaling pathways were enriched but no apoptosis pathway. Quantitative PCR results revealed the significantly higher mRNA level of *IL1B* and *CD69* in fresh NP-treated groups compared to that of aged ZnO NP- and zinc chloride-treated groups. The above results indicated that the lower cytotoxicity of aged ZnO NPs is partially attributed to their reduced potency in inducing apoptosis. The transcriptional regulation of multiple signal pathways activated by aged NPs may help to build the cellular homeostasis. Taken together, our findings highlight the influence of aging (environmental transformation) process of ZnO NPs on their toxicities and biological consequences.

## Introduction

With the rapid development of nanotechnology over the past decades, nanoparticles (NPs) have been applied in various fields, including industry, human daily life and nanomedicine [1, 2]. The Nanotechnology Consumer Product Inventory (CPI) shows a 30-fold increase between 2005 and 2015 in the numbers of nano-products, including 762 health (fitness) products, 72 food (beverages) and 23 baby products [2]. The growing application of NPs in consumer products and various fields increased the possibility of NPs entering into the environment, which raises safety concerns with regard to their potential adverse impacts. Zinc oxide (ZnO) NPs are among the most commonly-used NPs, and its global annual output has reached nearly 3400 tons [3, 4]. Some substances that are previously considered as biologically inert could become toxic in their nanoparticulate state. An increasing number of studies elucidated that ZnO NPs may pose significant risks to mammalian cells and animals by inducing significant toxicity [5–7].

Various strategies including coating, surface functionalization and oxidation state modification have been used to attenuate the potential toxicity of NPs by modifying the physical and chemical properties of them (such as the dissolution, agglomeration and perturbation of cell membranes) [8–11]. Although these modifications of NPs weaken their toxic effects in certain degree, the uses of NPs are not always safe, especially under certain exposure conditions and environments [12–14]. Actually, many kinds of NPs are not stable and inclined to undergo “aging” or “environmental transformation” after being intentionally or unintentionally released into the natural environment [14–17]. In recent years, lots of work were carried out to explore the environmental transformation process of NPs; however, the research on the toxic effects of “transformed (aged)” NPs is still very limited, let alone their toxic mechanisms.

As the typical representative of non-persistent NPs, ZnO NPs have very high reactivity, and are prone to transform in physical and chemical properties and occurrence state after being released into the environment or ingested by animals, which could significantly affect their toxicological effects [17, 18]. For example, studies have shown that the sulfidation process of ZnO NPs changed the charge, hydrophobicity and aggregation state, resulting in the adsorption of sulfide state NPs in human saliva, sweat and bronchoalveolar lavage fluid. Furthermore, the protein adsorbed by ZnO NPs forms a special protein crown that usually affects its biological effect [19]. Phosphates in physiological solutions could convert ZnO NPs into metastable ZnHPO_4_ and Zn_3_(PO_4_)_2_within about 5–10 h [20]. The process of complete transformation of ZnO NPs (≤ 3 μg/mL) in the in vitro exposure system to human T lymphocytes (37 °C, cell culture medium RPMI1640 containing 10% FBS for 24 h) was investigated by using synchrotron radiation X-ray absorption near-edge structure spectroscopy (XANES) [21]. The above studies suggest the underestimation of ZnO NPs’ environmental and health risks by solely evaluating the biological effects of pristine (fresh) ZnO NPs. In the light of this problem, there is an urgent need to comprehensively understand the aging and environmental transformation processes of NPs [22].

Our previous study revealed that ZnO NPs aged for 40–120 days in ultrapure water undergo physicochemical transformation and turn into Zn_5_(CO_3_)_2_(OH)_6_, Zn (OH)_2_, and Zn^2+^ [23]. Interestingly, aged ZnO NPs exhibited lower cytotoxicity than the fresh counterparts [23], yet the toxicity mechanisms of such kind of variation are unclear. In the present study, we set out to explore the underlying reasons of different cytotoxicities between fresh and aged ZnO NPs. ZnO NPs with two different particle sizes (20 nm and 90–200 nm) were applied systematically. The cytotoxicity assays demonstrated that aged ZnO NPs induced less pronounced morphological abnormalities and relatively higher cell viabilities than their fresh counterparts. RNA sequencing data revealed that apoptotic genes were enriched in fresh ZnO NP-treated cells, whereas these genes were much less affected by aged ZnO NP-treatments. In addition, the cells exposed to aged ZnO NPs showed reduced level of cleaved Caspase-3 protein, further indicating the higher potency of fresh ZnO NPs in eliciting apoptosis in cultured cells. Combined with our previous findings, this study suggested that the decreased cytotoxicity of aged ZnO NPs is attributed to their attenuated ability in triggering cell apoptosis.

## Materials and Methods

### Nanoparticles and Reagents

The commercially available ZnO nanopowders (ZnO NPs), with manufacturer’s reported average size 20 nm (99.5% purity, nearly spherical) and 90–200 nm (99.9% purity, irregular morphology), were purchased from Nanostructured & Amorphous Materials (Houston, TX). Except for otherwise noted, all the reagents and chemicals used in this study were purchased from Sigma-Aldrich (Shanghai, China).

### Nanoparticle Dispersion, Aging and Characterization

ZnO NPs stock suspensions (1 mg/mL) were prepared by suspending dry nanopowders in Milli-*Q* H_2_O (Millipore, 18 MΩ cm) and sterilized by autoclaving (120 °C, 30 min) and then stored at 25 °C for natural aging period ranging from 0 to 60 days. The 0- and 60-days’ naturally transformed ZnO NPs were designated as fresh and aged NPs, respectively. To ensure proper dispersion, the fresh and aged suspensions were sonicated (100 W) for 30 min in an ultrasonic bath before characterization or incubation with cells. The morphology, particle size and aggregation of fresh and aged ZnO NPs were characterized by using transmission electron microscopy (TEM, JEOL JEM-2010, Tokyo, Japan). The crystal structure of fresh and aged ZnO NPs was determined using power X-ray diffraction (XRD, PANalytical B. V., Shanghai, China) by comparing to authentic standards. The details of natural aging process and characterization on ZnO NPs have been described previously [23].

### Cell Culture and Treatment with ZnO NPs

*A*_L_ cell line, a kind of human–hamster hybrid cells formed by fusion of the *gly2A* mutant of Chinese hamster ovary (CHO) and human fibroblasts was used in this study. These hybrid cells contained a standard set of CHO-K1 chromosomes and a single copy of human chromosome 11 and were cultured in Ham’s F12 medium (Hyclone, Grand Island, NY) supplemented with fetal bovine serum (8%, Hyclone, Grand Island, NY), gentamicin (25 g/mL) and glycine (2 × 10^–4^ M) at 37 °C in a humidified 5% CO_2_ incubator [24]. The stock suspensions of fresh and aged ZnO NPs were dispersed by 30 min of ultrasonication (100 W) to prevent agglomeration, subsequently diluted to appropriate concentrations with cell culture media for the exposure of cells. Cells maintained in cell culture media without NPs were served as control in each experiment.

### Assay for Detecting the Cytotoxicity

*A*_L_ cells at a logarithmic phase of growth were cultured on glass slides in 35-mm Petri dishes (6 × 10^4^ cells/dish) for 24 h before stimulation, followed by treatment with 2 mL of culture medium containing 1, 5, 10, 12, 15 and 20 µg/mL fresh or aged ZnO NPs 72 h. After the completion of treatment time, the images of cell morphology were obtained using a Leica DM4B microscope (Leica, Germany). ZnCl_2_ was included as zinc ions reference for comparing the cytotoxicity with ZnO NPs.

The cell counting kit (CCK-8) (APExBIO, Shanghai, China) was used for detecting the cell viability. In details, *A*_L_ cells were seeded into 96-well plates (4 × 10^3^ cells/well) with cell culture media for 24 h and treated with medium containing various concentrations of ZnCl_2_, fresh and aged ZnO NPs for 24, 48 and 72 h, respectively. For working solution, the volume of added NPs from the stock suspension was less than 5% of the total volume of the culture medium in each well. After the completion of treatment time, the culture medium was aspirated, and the cells were incubated with 100 µL CCK-8 working solution for 2 h at 37 °C following the manufacturer’s instructions. Then, the absorbance was recorded at 450 nm using a Spectra Max M2 fluorescence reader (Molecular Devices, Wokingham, Berks, UK). Cell viability was calculated as a percentage of absorbance in wells, with each concentration of NPs normalized to the absorbance of control cells (100%).

### RNA Extraction, Reverse Transcription and Quantitative PCR

*A*_L_ cells at a logarithmic phase of growth were seeded into 35-mm-diameter Petri dish (6 × 10^4^ cells/dish) with cell culture media for 24 h. Then, the medium was replaced with 2 mL of culture medium containing 12 µg/mL ZnCl_2_, fresh and aged ZnO NPs for 72 h. After the completion of treatment time, the culture medium was aspirated, and cells were washed 3 times with PBS. Subsequently, 1 mL of Trizol reagent (Invitrogen, Carlsbad, CA, USA) was added to each dish to extract total RNA according to the manufacturer’s instructions. Concentration and purity of total RNA obtained after the extraction were quantified using a Q5000UV-Vis Spectrophotometer (Quawell, USA). After quantification, reverse transcription was performed using TransGene RT-PCR kit (TransGene Biotech, Beijing, China) to obtain cDNA from the RNA template according to the manufacturer’s protocols. The resulting cDNA samples were quantified by using the Q5000 UV–Vis Spectrophotometer and then analyzed using SYBR-Green as fluorescence dye (TransGene Biotech, Beijing, China) on Roche RT-PCR system (Applied Biosystems) [25].

The housekeeping gene encoding Glyceraldehyde-3-phosphate Dehydrogenase (*Gapdh*) was used as internal control for evaluating*Il-1α*, *Il-1β*, *Caspase 3*, *CD69*, *Jun* and *MT1* mRNA expression. The results were expressed as the relative expression ratio between the targeted gene and *Gapdh*. The primer sequences used in this study are provided in Table 1.

**Table 1:** Primers used in this study

Name	Primer	Sequence	Length (bp)
*Il-1α*	Forward	5′CGTCCGTCGTAATATCAG3′	178
	Reverse	5′GACTTCATGGGACGATATG3′	
*Il-1β*	Forward	5′ACCTTCCAGGATGAGGACATGA3′	121
	Reverse	5′CTAATGGGAACGTCACACACCA3′	
*Caspase 3*	Forward	5′CACATGTTCTCTGGGAAATCG3′	162
	Reverse	5′TTGTATCTCTGGAAGTTTCAGATTGTT3′	
*CD69*	Forward	5′GCCACCACGCTCTTCTGTCTAC3′	163
	Reverse	5′GGGTCTGGGCCATAGAACTGAT3′	
*Jun*	Forward	5′CACATGTTCTCTGGGAAATCG3′	177
	Reverse	5′TTGTATCTCTGGAAGTTTCAGATTGTT3′	
*MT1*	Forward	5′GCCACCACGCTCTTCTGTCTAC3′	126
	Reverse	5′GGGTCTGGGCCATAGAACTGAT3′	
*Gapdh*	Forward	5′GTTAAGCAGTACAGCCCCAAA3′	123
	Reverse	5′AGGGCATATCCAACAACAAACTT3′	

### RNA Sequencing Data Analysis

The total RNA samples of *A*_L_ cells from control group, aged ZnO NP-treated group and ZnCl_2_ treated group were sequenced by BangFei Bioscience (Beijing, China). Briefly, the total RNA of *A*_L_ cells was extracted following the TRIZOL protocols, until the isoproponal precipitation. Then, the RNA samples were resuspended in the extraction buffer before sequencing. The raw count RNA sequencing data were analyzed using R package Deseq2 [Eric1]. The venn diagram was generated by R package VennDiagram [Eric1.2]. The significantly changed genes were used for further pathway enrichment analysis. Experiments were done three independent replicates. rRNA genes, mitochondrial genes and the genes detected less than 40 bp were excluded from the analysis.

The RNA sequencing data, reference series GSE97852, GSE60159 and GSE39444, were obtained from Gene Expression Omnibus [Eric 2, 3, 4]. The Gene Set Enrichment Analysis plot was generated by R (version 3.6.2) using package fgsea [Eric 5]. The apoptosis genes with 1.5-fold significant change & *p* value < 0.05 were used for further analysis. The heatmap with gene tree was generated by R package “ComplexHeatmap” [Eric 6]. Average linkage was used as the clustering method, and Euclidean was used as a distance measurement method. The pathway enrichment analysis was preceded using STRING2.0 [Eric 7].

### Western Blotting

*A*_L_ cells at a logarithmic phase of growth were seeded into 60-mm-diameter Petri dish (1.5 × 10^5^ cells/dish) with cell culture media for 24 h. Then, the medium was replaced with 4 mL of culture medium containing 12 µg/mL fresh or aged ZnO NPs for 24 h. At the end of exposure period, the culture medium was aspirated, and then, cells were washed 3 times with PBS and lysed on ice with RIPA lysis buffer (Beyotime, China) to collect cellular proteins. Equal amounts of cellular proteins were separated on 12% SDS-PAGE gels and then transferred to a polyvinylidene fluoride (PVDF) membrane (Roche, Swiss). Briefly, after 2 h blocking with 5% nonfat milk in TBST at 25 °C, the membranes were subsequently incubated with primary antibody at appropriate dilutions (according to the manufacturer’s protocols) at 4 °C overnight, followed by incubating with HRP-conjugated secondary antibodies (1:5000, Promega, Madison, USA) for 2 h at 25 °C. Finally, immunolabeling was detected using an enhanced chemiluminescence (ECL) (BOSTER, China) solution. The primary antibodies of anti-pro/cleaved Caspase-3 and anti-Actin were purchased from Cell Signaling Technology and ImmunoWay, respectively.

### Statistics

Statistical analysis was compiled on the means of the results obtained from at least three independent experiments. All Data were presented as means ± standard deviation (SD) and statistically compared using one-way analysis of variance (ANOVA). In all plots *p* values < 0.05 were showed as * and considered to be statistically significant.

## Results

### Characterization of ZnO NPs

**Fig. 1:**
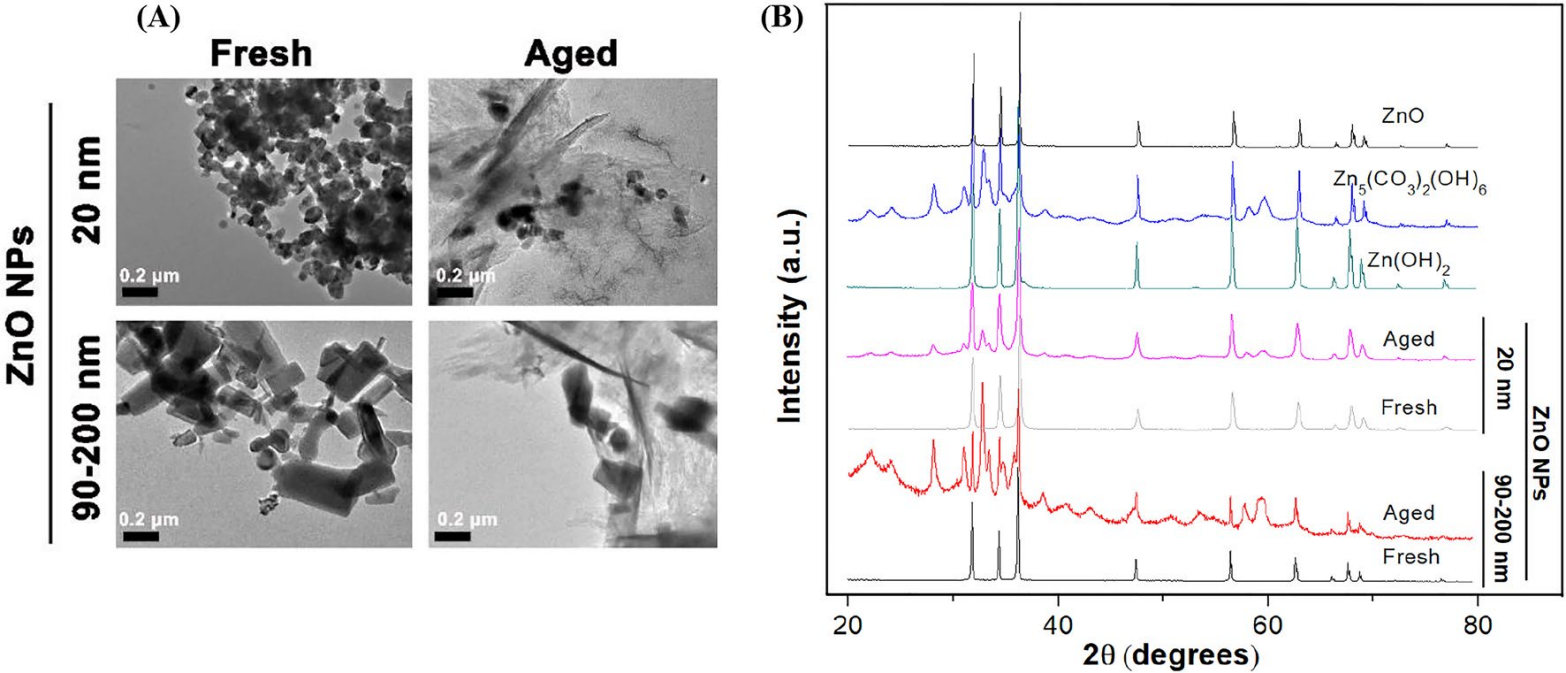
Physicochemical characteristics of fresh and aged ZnO NPs. **A** Representative micrographs of fresh and aged NPs (100 μg/mL, 20 and 90–200 nm) in Milli-*Q* water using low resolution TEM, **B** XRD patterns of fresh NPs, aged NPs, ZnO, Zn (OH)_2_ and Zn_5_(CO_3_)_2_(OH)_6_ references in dried form

To determine the differences in detailed physicochemical characteristics between fresh and aged ZnO NPs, we first observed the morphology of NPs using TEM (Fig. 1A). Our results indicated that 20 nm fresh ZnO NPs were nearly spherical crystals and 90–200 nm fresh ZnO NPs were irregularly rod-like/cubical crystals. The single particle size was consistent with the size provided by the manufacturer. Obviously, both 20 nm and 90–200 nm ZnO NPs were inclined to aggregate in ultrapure water. Also, regardless of the shape and size of the original NPs, both 20 nm and 90–200 nm ZnO NPs’ microstructure was dramatically changed from a clear crystal structure to an amorphous or sheet-/needle-like state after aged for 60 days. Furthermore, the crystalline nature and phase purity of both fresh and aged NPs were determined by using X-ray diffraction (XRD) with Cu Kα radiation (*λ* = 0.15418 nm) approach at 25 °C, as shown in Fig. 1B. The XRD pattern of fresh ZnO NPs indicated that the samples were comprised of crystalline wurtzite structure and no characteristic impurity peaks were identified, suggesting a high quality of fresh NPs. For aged NPs, the XRD pattern exhibited the neoformation of Zn_5_(CO_3_)_2_(OH)_6_ (card number 00-011-0287) and Zn (OH)_2_ (card number 00-003-0888) solid phases, indicating the chemical transformation of ZnO NPs (20 and 90–200 nm) during the aging process.

### Morphological Observation of ***A***_L_ Cells Exposed to Fresh and Aged ZnO NPs

**Fig. 2:**
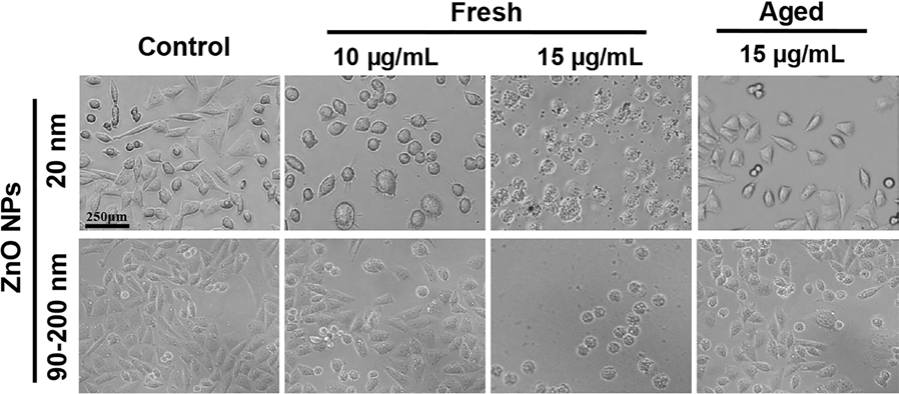
Morphology changes in *A*_L_ cells after exposure to fresh or aged ZnO NPs for 72 h in supplemental Ham’s F12 medium, and unexposed cells were used as control groups. *A*_L_ cell morphology was observed with an optical microscope at 10 × magnification

NPs’ treatment results in a noticeable change in cellular shape, or morphology, in vitro [26]. Therefore, *A*_L_ cells exposed to fresh or aged ZnO NPs at 10 & 15 µg/mL for 72 h were examined under a stereoscopic microscope. As shown in Fig. 2, cell morphology in the control group remained normal. The cells adhered well, with most attaching within 2 h. Most cells were spindle shaped or polygonal, with a few newly dividing cells showing a more transparent cytoplasm and better dispersion during the process of adhering. Treatment with 12 μg/mL fresh ZnO NPs (20 nm & 90–200 nm) for 72 h significantly changed cell morphology. Although most cells adhered within 3–5 h, they could not spread well, and some cells became rounded and lost the polygonal shape. When the concentration of ZnO NPs was increased to 15 μg/mL, the treated cells atrophied and could not adhere, suggesting their significantly lower cell viability than that of the cells treated with 10 μg/mL. These results indicated that the LC100 for fresh ZnO NPs is probably less than 15 μg/mL by a 72-h treatment. In contrast, cell morphology in 20 nm and 90–200 nm aged NP-treated groups (15 μg/mL) was not significantly affected, and most of the surviving cells could adhere and spread, with less than half dead cells observed, unveiling that aged ZnO NPs are much lower cytotoxic than fresh ZnO NPs.

### Aged ZnO NPs Induced Lower Cytotoxicity than Fresh NPs

**Fig. 3:**
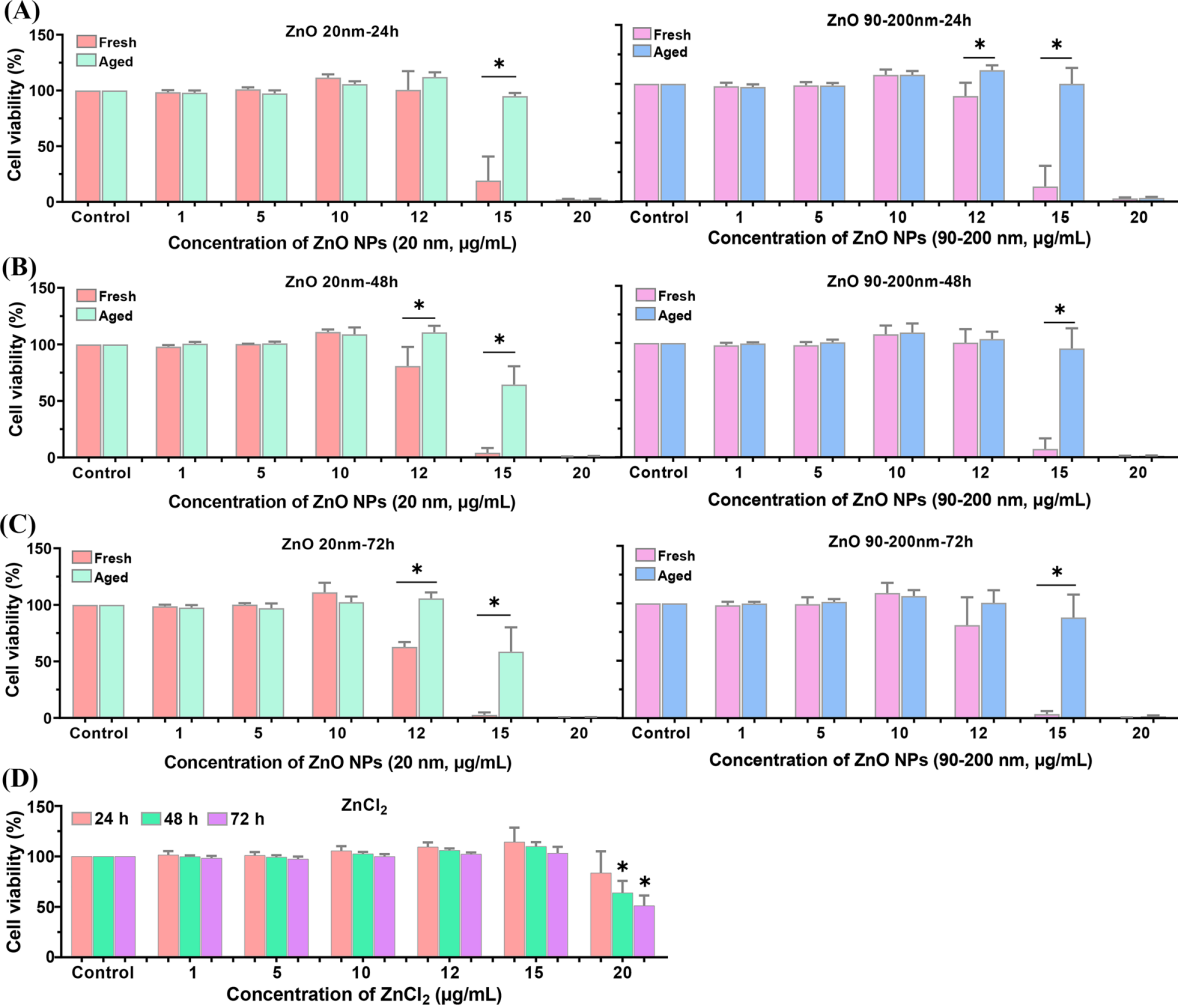
Cell viability induced by fresh and aged ZnO NPs in *A*_L_ cells. *A*_L_ cells were incubated with various concentrations of fresh and aged ZnO NPs (20 and 90–200 nm) for 24 h (**A**), 48 h (**B**) and 72 h (**C**). **D** *A*_L_ cells were exposed to various concentrations of ZnCl_2_ for different times. Data were based on ≥ 3 independent experiments and expressed as mean ± SD, *p < 0.05

To further investigate the difference in cytotoxicity between fresh and aged ZnO NPs, we examined the cell viability by using CCK-8 kits. As shown in Fig. 3, incubation *A*_L_ cells with gradient doses of fresh and aged ZnO NPs (ranging from 0 to 20 μg/mL, 20 nm and 90–200 nm) for 24 h, 48 h or 72 h showed a dose-dependent decrease in cell viability. No obvious cytotoxicity was observed by treating cells with ZnO NPs ≤ 10 μg/mL. When the dosage of fresh and aged ZnO NPs elevated to 12 and 15 μg/mL, the cell viability showed a time-dependent decrease tendency. Obviously, the cell viability in aged NP-treated groups was significantly higher than fresh NP-treated groups. In addition, ZnCl_2_-treatment also compromised cell viability in a dose- and time-dependent manner, whereas the cytotoxicity of ZnCl_2_ was much less than that of both fresh and aged ZnO NPs.

### Fresh ZnO NPs’ Treatment Activated Apoptosis Pathways and Up-Regulated the Expression of Apoptotic Genes

**Fig. 4:**
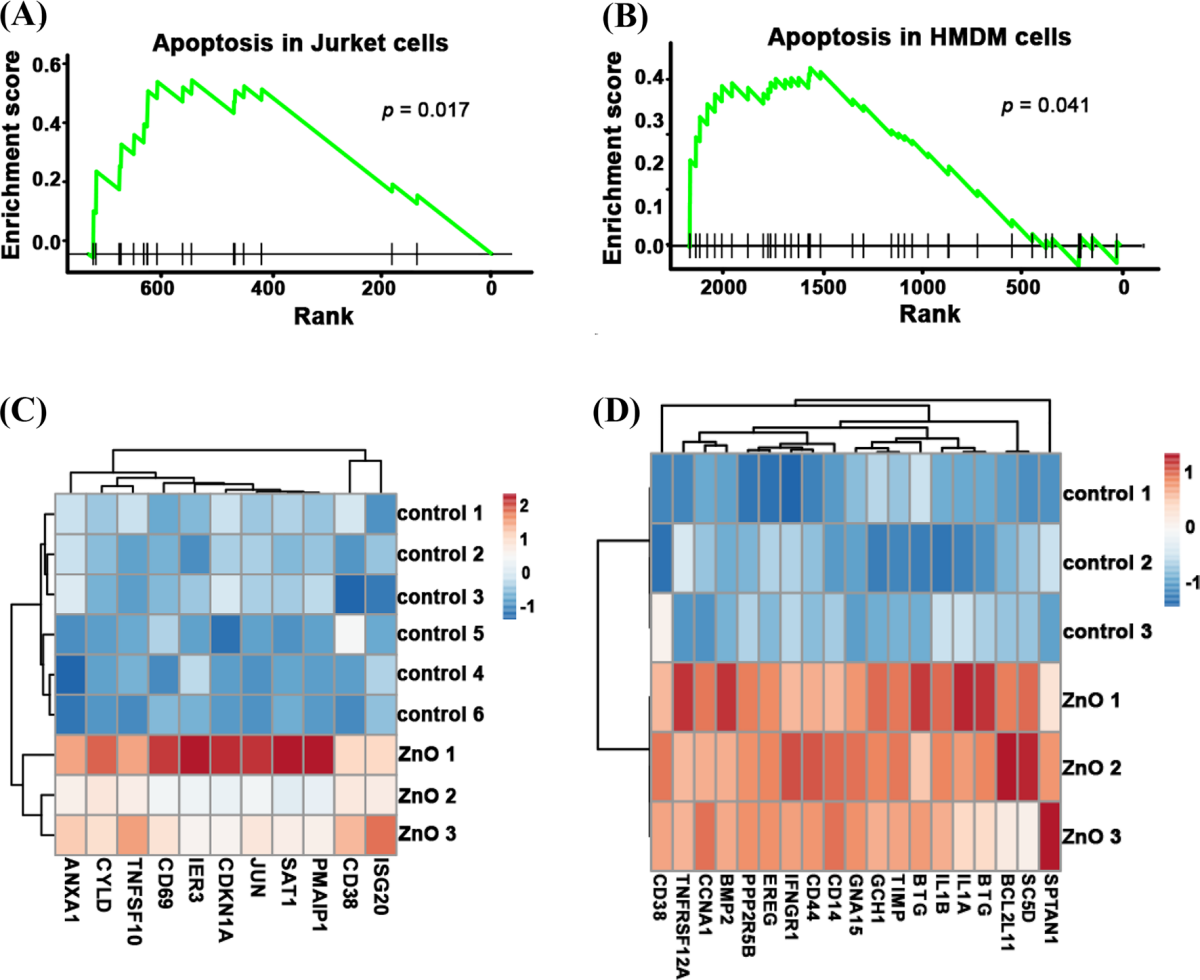
Apoptosis pathway was enriched in RNA-seq data of fresh ZnO NP-treated Jurket and HMDM cells. The enrichment score of significantly expressed genes from apoptosis pathway of fresh ZnO NP-treated Jurket cells (**A**) and HMDM cells (**B**). The heatmap of apoptotic gene expression of fresh ZnO NP-treated Jurket cells (**C**) and HMDM cells (**D**) and their control groups

To unveil the underlying mechanisms leading to the lower cytotoxicity of aged NPs, we analyzed RNA sequencing data from both fresh and aged ZnO NPs. As shown in Fig. 4A, B, after treatment with fresh ZnO NPs, apoptosis pathway was activated in Jurkat cells (*p* = 0.017) and HMDM cells (*p* = 0.041). The apoptosis genes: *ANXA1*, *CYLD*, *TNFSF10*, *IER3*, *CDKN1A*, *JUN*, *SAT1*, *PMAIP1*, *CD38* and *ISG20* were significantly enriched in fresh ZnO NP-treated Jurkat cells. The apoptosis genes: *CD38*, *TNFRSF12A*, *CCNA1*, *BMP2*, *PPP2R5B*, *EREG*, *IFNGR1*, *CD44*, *CD14*, *GNA15*, *GCH1*, *TIMP1*, *BTG2*, *IL1B*, *IL1A*, *BTG3*, *BCL2L11*, *SC5D* and *SPTAN1* were significantly enriched in fresh ZnO NP-treated HMDM cells (Fig. 4C, D). Since Jurket cells (peripheral blood T lymphocyte cells) and HMDM cells (human monocyte-derived macrophages) are different kinds of cells, the way they trigger apoptosis might be different. In sum, these results showed that fresh ZnO NPs’ exposure could activate different apoptosis pathways in various kinds of cells.

### Aged ZnO NPs Did Not Up-Regulate the Expression of Apoptotic Genes as Fresh ZnO NPs

**Fig. 5:**
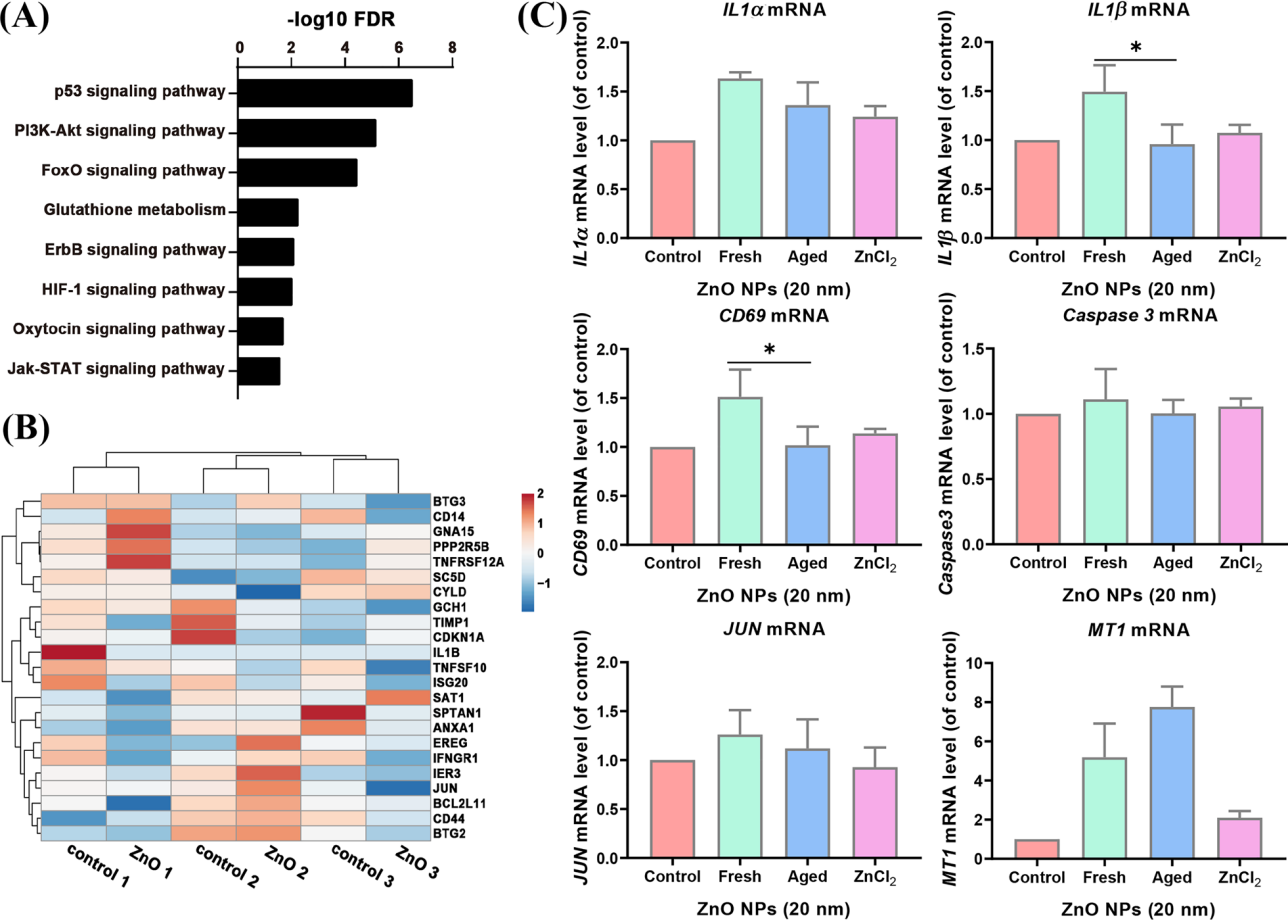
Apoptosis pathway was not enriched in RNA-seq data of aged ZnO NP-treated *A*_L_ cells. (**A**) The gene ontology analysis of enriched pathways from aged ZnO NP-treated *A*_L_ cells. (**B**) The heatmap of apoptotic gene expression of aged ZnO NP-treated *A*_L_ cells and control group. (**C**) The expression of selected apoptotic genes and control genes (*MT1*) in fresh and aged ZnO NP-treated *A*_L_ cells

Our RNA sequencing data from aged ZnO NP-treated *A*_L_ cells showed that p53, PI3k–Akt, FoXO, Glutathione, ErbB, HIF-1, Oxytocin and Jak-STAT signaling pathway were enriched (Fig. 5A). The apoptosis genes enriched in Jurket and HMDM cells were not significantly affected in the aged ZnO NP-treated cells (Fig. 5B). To further confirm the findings, we tested the expression of related genes by real time PCR. We found that some of the apoptosis genes: *BMP2*, *PMAIP1*, *IL1α*, *CD69*, *CCNA1*, *CD38* and *IL1β* were undetectable in aged ZnO NPs-treated *A*_L_ cells (data not shown), probably because most of these genes are expressed in immune system cells. The other up-regulated apoptosis genes (*IL1α*, *IL1β* and *CD59*) observed in fresh ZnO NP-treated groups were not significantly changed in expression levels by aged ZnO NPs’ treatment. While the *MT1* that serve as a positive control was significantly increased in an expression level, the expression of *Caspase 3* was not significantly changed (Fig. 5C). These data suggested that aged ZnO NPs, unlike their fresh counterparts, are less potent in activating apoptosis pathway genes in *A*_L_ cells.

### Fresh But Not Aged ZnO NPs Increased the Expression Level of the Activated Caspase 3 Protein

**Fig. 6:**
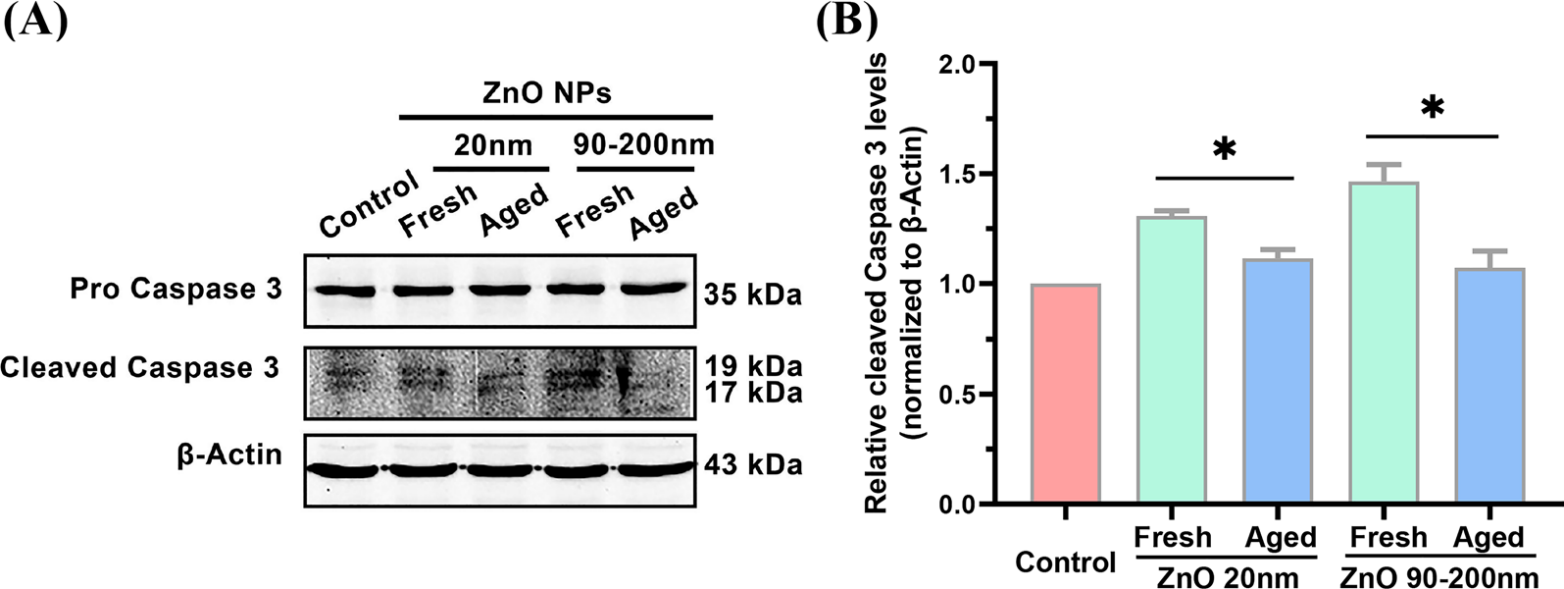
Apoptotic levels in *A*_L_ cells incubated with fresh and aged ZnO NPs (20 and 90–200 nm). Western Blotting analysis (**A**) and quantification (**B**) of cleaved Caspase 3 protein levels when cells were incubated with 12 μg/mL fresh and aged ZnO NPs (20 and 90–200 nm) for 72 h. Data were based on ≥ 3 independent experiments and expressed as mean ± SD, *p < 0.05

Detection of gene expression of *Caspase 3* alone cannot directly indicate the activation of apoptosis pathway. To further analysis whether ZnO NPs’ treatment could change apoptotic proteins’ level, the expression of cleaved Caspase 3 protein, a commonly used biomarker to indicate the activation of cell apoptosis [27], was examined by Western blotting analysis. As shown in Fig. 6, compared to the control group, fresh ZnO NPs (20 nm) treatment increased the cellular level of cleaved Caspase 3 protein by 1.31 ± 0.023-fold, which was significantly higher than that of aged 20 nm ZnO NPs-treated group (1.12 ± 0.039-fold). When the particle size of fresh ZnO NPs was increased to 90–200 nm, the expression of cleaved Caspase 3 protein induced by fresh NPs was increased by 1.46 ± 0.078-fold, significantly greater than that of aged NPs (1.07 ± 0.075-fold). These data further illustrated the higher potency of fresh ZnO NPs in inducing cell apoptosis, in comparison with their aged counterparts.

## Discussion

ZnO NPs were reported to undergo physicochemical transformation into Zn_5_(CO_3_)_2_(OH)_6_ with the release of Zn^2+^ during the natural aging process [23, 28]. However, the cytotoxicity induced by the transformed (aged) ZnO NPs and the underlying mechanisms remain unclear. Here, to unveil the mechanism of diverse cytotoxicity between fresh and aged ZnO NPs, RNA sequencing analysis and RT-PCR test were conducted. Also, Western blotting was applied to examine the protein level of Caspase 3, the key executor in cell apoptosis.

Our data showed that aged ZnO NPs induced much less cytotoxicity than fresh ZnO NPs in *A*_L_ cells. The LC_100_ of both fresh ZnO NPs (90–200 nm and 20 nm) in our present study was lower than 15 μg/mL (Fig. 3), which was consistent with previous findings that the LC_100_ of ZnO NPs with 19–36 nm to NIH-3T3 or MSTO cell is about 15 μg/mL [29]. We confirmed that the environmental transformations of physicochemical properties in NPs can dramatically alter their toxicity. It has been reported that the sulfidation process of ZnO NPs changes their charge, hydrophobicity and aggregation state, resulting in the adsorption of sulfide state NPs in human saliva, sweat and bronchoalveolar lavage fluid. And the protein adsorbed by ZnO NPs formed a special protein crown, which affected its biological effect [19]. Phosphates widely present in physiological solutions (such as saliva) could convert ZnO NPs into metastable ZnHPO_4_ and Zn_3_(PO_4_)_2_ within about 5–10 h and showed cytotoxicity to digestive tract epithelial cells [20]. Ivask et al. proved the occurrence of complete transformation of ZnO NPs (≤ 3 μg/mL) in the in vitro exposure system to human T lymphocytes (37 °C, cell culture medium RPMI1640 containing 10% FBS for 24 h) using synchrotron radiation X-ray absorption near-edge structure spectroscopy (XANES). The spectrum and cytotoxicity of the transformation products were consistent with those of ZnSO_4_ [21]. Our results revealed the dose- and time-dependent toxicity of ZnCl_2_ to *A*_L_ cells, where its cytotoxicity is much lower than both fresh and aged ZnO NPs (Fig. 3). The observation further explains the finding that the cytotoxicity of fresh ZnO NP is not fully attributed to its released Zn^2+^ [30].

**Fig. 7:**
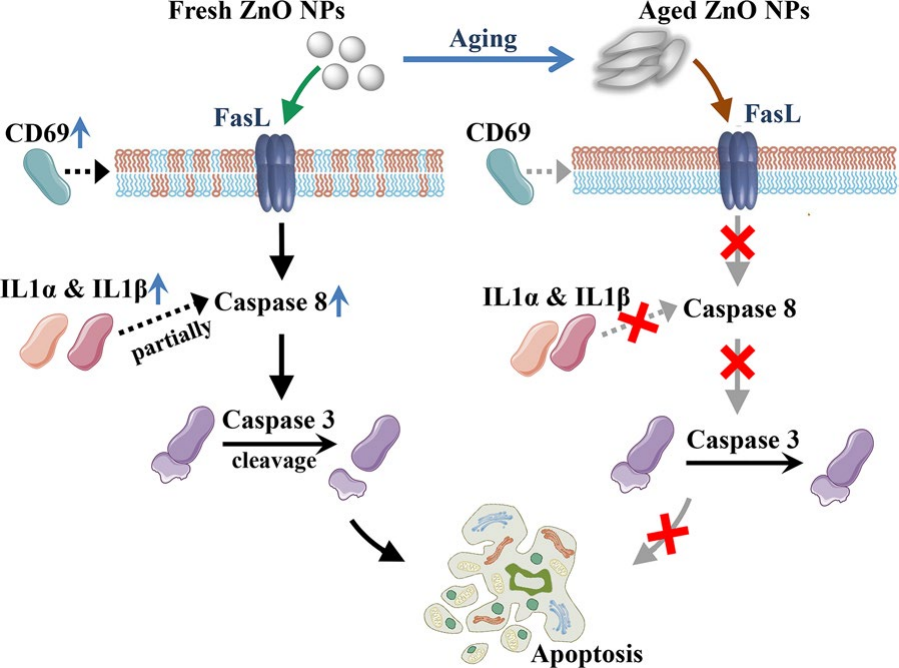
Model for Fresh ZnO NPs but not aged ZnO NPs induces Caspase 8- and Caspase 3-dependent apoptosis. The increased expression of apoptotic gene *CD69* activates Fas and apoptosis annexin V expression in fresh ZnO NP-exposed mammalian cells. The increased expression of apoptotic gene *IL1α* and *IL1β* partially activates Caspase 8-dependent apoptosis. It further causes activation of Caspase 3 and induces apoptosis. All these changes in mRNA and protein level were not detectable in aged ZnO NPs-exposed mammalian cells

Our previous study also showed that aged ZnO NPs exhibit a higher potency in eliciting ROS (reactive oxygen species), as well as an attenuated ability in killing cells compared to fresh ZnO NPs [23]. We reason that the lower cytotoxicity induced by aged ZnO NPs could be more tolerable in mammalian cells. The present study of RNA sequencing data illustrated that apoptotic genes have been up-regulated in fresh ZnO NP-treated cells, where they were much less affected in aged NP-treated groups. IL1α and IL1β are members of the interleukin 1 cytokine family. The release of IL1α and IL1β activates Caspase 8 partially dependent apoptosis [31]. CD69 encodes a member of the calcium-dependent lectin superfamily of type II transmembrane receptors. Increased CD69 expression was associated with an increased expression of the apoptosis annexin V and CD95 (Fas) marker [32]. JUN is an AP-1 transcription factor subunit. Increased JUN activity proteolytically cleavages alpha-fodrin, a substrate of the interleukin 1beta-converting enzyme (ICE), and CED-3 family of cysteine proteases, which further causes programmed cell death [33]. The increased expression of these apoptotic genes revealed that fresh NPs trigger apoptosis in several different ways. After the elevation of these apoptotic gene expressions, apoptosis processes are eventually executed by apoptotic proteins (Fig. 7). Caspase 3 is the core protease for various apoptotic scenarios; cleavage of this protein is necessary to activate both extrinsic and intrinsic apoptotic pathways [34, 35]. Therefore, detection of cleaved caspase 3 is a common method for identifying apoptosis induced by a wide variety of apoptotic signals [36]. Our Western blotting data revealed that, for both 20 nm and 90–200 nm ZnO NPs, sublethal exposure did not alter the level of Pro caspase 3 in all treatment groups. In contrast, cleaved Caspase 3 was significantly elevated by fresh NPs treatment, where aged NPs showed few (if any) effects on the level of cleaved caspase 3 (Fig. 6). Combined with RNA expression analysis, our results clearly elucidated the higher potency of fresh ZnO NPs in inducing cell apoptosis.

## Conclusions

In the present study, the natural physicochemical transformation of ZnO NPs in ultrapure water was confirmed, and variations in cytotoxicity induced by fresh & aged NPs were investigated. We focused on RNA sequencing data from our aged ZnO NP-treated *A*_L_ cells and that of fresh NPs from database. We compared those signaling pathway specifically enriched in aged NP-treated group, which are different from that of fresh NP- or ZnCl_2_-treated groups. Our data indicated that the lower cytotoxicity of aged ZnO NPs is closely related to its attenuated ability in inducing apoptosis, while the transcriptional regulation of the multiple pathways activated by NPs promotes the establishment of cellular homeostasis in mammalian cells.

## Data Availability

Not applicable.

## Abbreviations

NPs: Nanopowders
ZnO: Zinc oxide
Zn_5_(CO_3_)_2_(OH)_6_: Hydrozincite
Zn (OH)_2_: Zinc hydroxide
ZnCl_2_: Zinc chloride
ZnSO_4_: Zinc sulfide
ZnHPO_4_: Zinc hydrogen phosphate
Zn_3_(PO_4_)_2_: Zinc phosphate
*A*_L_ cells: Human–hamster hybrid cells
CHO cells: Chinese hamster ovary cells
Jurket cells: Peripheral blood T lymphocyte cells
HMDM cells: Human monocyte-derived macrophages
NIH-3T3cells: Mouse embryonic cells
MSTO cells: Human lung cancer cells
RPMI1640: Roswell Park Memorial Institute 1640
ICE: Interleukin 1beta-converting enzyme
CED-3: *Caenorhabditis elegans* death gene
IL1α: Interleukin 1alpha
IL1β: Interleukin 1beta
mRNA: Messenger ribonucleic acid
cDNA: Complementary deoxyribonucleic acid
FBS: Fetal bovine serum
TEM: Transmission electron microscopy
XRD: X-ray diffraction
RT-PCR: Real-time polymerase chain reaction
CPI: The Nanotechnology Consumer Product Inventory
XANES: Synchrotron radiation X-ray absorption near-edge structure spectroscopy
RIPA: Radio immunoprecipitation assay
SDS-PAGE: Polyacrylamide gel electrophoresis
PVDF: Polyvinylidene fluoride
ECL: Enhanced chemiluminescence
CCK-8: Cell counting kit-8
